# Hyperæmia of the Conjunctiva — Simple Conjunctivitis

**Published:** 1892-05-14

**Authors:** 


### OPHTHALMOLOGY.

### HYPERÆMIA OF THE CONJUNCTIVA—SIMPLE CONJUNCTIVITIS.

By hyperæmia of the conjunctiva we mean what is commonly known as cold in the eye, being very often the result of exposure to a cold wind or draught, or coming on after getting a thorough soaking. It is also produced by the presence of a foreign body, such as a fly, a bit of dust, or a smut from a smoky chimney, in the conjunctival sac, as well as from irritating vapours, as ammonia, tobacco smoke, &c. It may be brought on by trying the sight too much, attempting to read in a bad light, by firelight, or by prolonged efforts at accommodation in eyes, either hyperopic or astigmatic, or where the muscles of the eye are defective. Too bright a light has been known to produce it, such as powerful electric light, and the reflection from snow when long continued. It may also be produced by inversion of the cilia, so that they rub against the ocular conjunctiva, and may accompany all diseases of the eyelids, cornea, or iris.

The hyperæmia may be confined to the ocular conjunctiva, or may be continued to the palpebral also. It also varies much in amount and severity, the gradation from hyperæmia to catarrhal conjunctivitis, with slight secretion of mucus, being very gradual, rendering it difficult to draw a hard and fast line between the more severe cases of hyperæmic conjunctivitis and the milder forms of catarrhal conjunctivitis.

The patient feels as if some dust had got into the eye, and can sometimes hardly be persuaded to the contrary. The white of the eye (the sclerotic) is covered by a network of small vessels situated in the conjunctiva, and these become injected according to the severity of the case, causing it to look pinkish, or quite red. These vessels being in the conjunctiva can be moved by the finger over the surface of the sclerotic, and if the lid be pressed on them from the corneal margin outwards, the vessels can be temporarily emptied, refilling as the pressure is taken off. This is a valuable diagnostic sign in distinguishing this conjunctival hyperæmia from the ciliary congestion around the margin of the cornea, seen in acute iritis, and which is situated in the sub-conjunctival vessels. It is also accompanied by a varying amount of photophobia and lachrymation.

The treatment consists in removing any cause of irritation, if present, and applying a soothing lotion. When the hyperæmia is due to the wound caused by a foreign body, the application of cocain in solution is serviceable both in relieving the pain, and also by causing the dilated vessels to contract. In cases arising from cold, some weak astringent locally applied will effect a cure. Alum sulphate 4 to 8 grs. to the ℥i of water is the simplest; sulphate of zinc, gr.i to the ℥i, tannin, &c., are also useful. A common household remedy is cold tea, which is probably useful from the solution of tannin it contains. A shade may be worn to protect the eyes from the light. When a patient has a prolonged attack, or recurrent attacks, the condition of the refraction should be inquired into, and suitable glasses prescribed.

Sometimes one of these dilated vessels gives way, and a subconjunctival hæmorrhage occurs, the whole of the surface of the sclerotic being covered, and the eye appearing bright red all over up to the edge of the cornea. This is most alarming to the patient, but it can safely be prognosed that the hæmorrhage will be absorbed in about ten days, and the eye will recover itself too. An iced pad to the eyelids, or a cocain, or astringent lotion to the conjunctiva to prevent further hæmorrhage may be recommended for the patient to apply in the meantime, and one often gets more thanks for curing this simple conplaint than for attention to some much more serious trouble.

